# Knee loading inhibits osteoclast lineage in a mouse model of osteoarthritis

**DOI:** 10.1038/srep24668

**Published:** 2016-04-18

**Authors:** Xinle Li, Jing Yang, Daquan Liu, Jie Li, Kaijun Niu, Shiqing Feng, Hiroki Yokota, Ping Zhang

**Affiliations:** 1 Department of Anatomy and Histology, School of Basic Medical Sciences, Tianjin Medical University, Tianjin 300070, China; 2 Department of Pharmacology, Institute of Acute Abdominal Diseases, Tianjin Nankai Hospital, Tianjin 300100, China; 3 Nutritional Epidemiology Institute and School of Public Health, Tianjin Medical University, Tianjin 300070, China; 4 Department of Orthopedics, Tianjin Medical University General Hospital, Tianjin 300052, China; 5 Department of Biomedical Engineering, Indiana University-Purdue University Indianapolis, IN 46202, USA; 6 TEDA International Cardiovascular Hospital, Chinese Academy of Medical Sciences & Peking Union Medical College, Tianjin 300457, China

## Abstract

Osteoarthritis (OA) is a whole joint disorder that involves cartilage degradation and periarticular bone response. Changes of cartilage and subchondral bone are associated with development and activity of osteoclasts from subchondral bone. Knee loading promotes bone formation, but its effects on OA have not been well investigated. Here, we hypothesized that knee loading regulates subchondral bone remodeling by suppressing osteoclast development, and prevents degradation of cartilage through crosstalk of bone-cartilage in osteoarthritic mice. Surgery-induced mouse model of OA was used. Two weeks application of daily dynamic knee loading significantly reduced OARSI scores and CC/TAC (calcified cartilage to total articular cartilage), but increased SBP (subchondral bone plate) and B.Ar/T.Ar (trabecular bone area to total tissue area). Bone resorption of osteoclasts from subchondral bone and the differentiation of osteoclasts from bone marrow-derived cells were completely suppressed by knee loading. The osteoclast activity was positively correlated with OARSI scores and negatively correlated with SBP and B.Ar/T.Ar. Furthermore, knee loading exerted protective effects by suppressing osteoclastogenesis through Wnt signaling. Overall, osteoclast lineage is the hyper responsiveness of knee loading in osteoarthritic mice. Mechanical stimulation prevents OA-induced cartilage degeneration through crosstalk with subchondral bone. Knee loading might be a new potential therapy for osteoarthritis patients.

Osteoarthritis (OA) is characterized by degeneration of articular cartilage and remodeling of the subchondral bone leading to functional impairment in synovial joints, affects approximately 630 million people in the world^1^,^2^. Structural changes such as the articular cartilage and subchondral bone uncontrolled metabolic processes of OA could be diagnosed by MRI, radiography or arthroscopy including probing techniques^3^,^4^,^5^,^6^. Nowadays, OA is considered as a whole-joint disease^7^. The degeneration of the cartilage can be heavily influenced by subchondral bone remodeling through crosstalk of bone-cartilage^8^,^9^. The cartilage and subchondral bone form a unit and play crucial roles in osteoarthritis pathogenesis^10^.

Cartilage degeneration, osteophyte growth, subchondral bone plate thinning, and subchondral bone cysts formation are important factors that contribute to OA histopathology, caused by dysregulation of osteoclast (OC) and osteoblast (OB) activity in subchondral bone^11^. Osteoclasts associate with loss of integrity and function at the cartilage and subchondral bone in OA. In previous studies, positive histological staining of tartrate-resistant acid phosphatase (TRAP) for the number of osteoclast in the subchondral bone increased in both OA animal and patients^6^,^12^,^13^,^14^, and TRAP enzyme activity highly increased in synovial fluid of OA^15^. Expression of mRNA levels of osteoclastic bone resorption markers including Cathepsin K and TRAP, were also elevated at 2 weeks after osteoarthritis induced by surgery^16^. Activated osteoclasts of OA bone promote cartilage degeneration by secreting various cytokines and growth factors^17^. But the relationship between osteoclast activity and pathobiological changes of OA has yet to be fully elucidated. Due to osteoclast activity is involved in the progression of osteoarthritis, suppressing abnormal osteoclast activity might be a strategy to prevent osteoarthritis.

Treatments of osteoarthritis include bio-mechanical interventions, oral agents, and surgery^18^. Oral agents increase risk of serious gastrointestinal disorder, cardiovascular and renal injury, and joint replacement surgery is executed in the end stage of OA. Bio-mechanical interventions, such as exercise and bracing increasingly combines with oral agents to be reduce pain and disability for OA^5^. Moderate pulsating mechanical loading which applied laterally dynamic loads to knee has been shown strengthening bone^19^. The loading force required to prevent degradation of cartilage due to knee loading is smaller than that of other mechanical loading regimens. Knee loading not only stimulates bone formation but also attenuates pain perception-linked signaling^20^,^21^,^22^,^23^, and MMP13 activity in the mouse cartilage is reduced by mechanical loading^24^. Furthermore, mechanical loading also altered Wnt/β-catenin signaling pathway in bone^25^,^26^. These characteristics make knee loading to be a potential treatment for osteoarthritis as a bio-mechanical intervention. However, the effect of knee loading on osteoclast activity of osteoarthritis is not completely clear.

We hypothesized that osteoclast lineage plays an important role in restoring the structure of subchondral bone and preventing cartilage degradation, and osteoclastogenesis is suppressed in osteoarthritis treatment by knee loading through stimulating Wnt signaling pathway. To test this hypothesis, a mouse model of osteoarthritis was induced by transecting medial collateral ligament and removing medial meniscus. The effects of knee loading on Osteoarthritis Research Society International (OARSI) scores, thickness of subchondral bone plate, activity of osteoclasts in subchondral bone, as well as formation, migration and adhension of osteoclasts from bone marrow-derived cells were evaluated in surgery-induced OA mouse model. The correlation between osteoclast activity and OARSI scores, parameters of subchondral bone was analyzed. Multiple linear regression models for osteoclast activity and OARSI scores, parameters of subchondral bone were also generated to evaluate the links between osteoclast activity and osteoarthritis histopathological changes. To evaluate the underlying mechanism, the expressions of Wnt3a in Wnt signaling pathway, together with osteoclastogenesis genes including Nuclear Factor of Activated T-cells cytoplasmic 1 (NFATc1), Receptor Activator for Nuclear Factor-κ B Ligand (RANKL), Tumor Necrosis Factor-α (TNF-α), and Cathepsin K were detected in knee loading effects on a mouse model of osteoarthritis.

## Results

### Histomorphological analysis of cartilage

**Figure 1:**
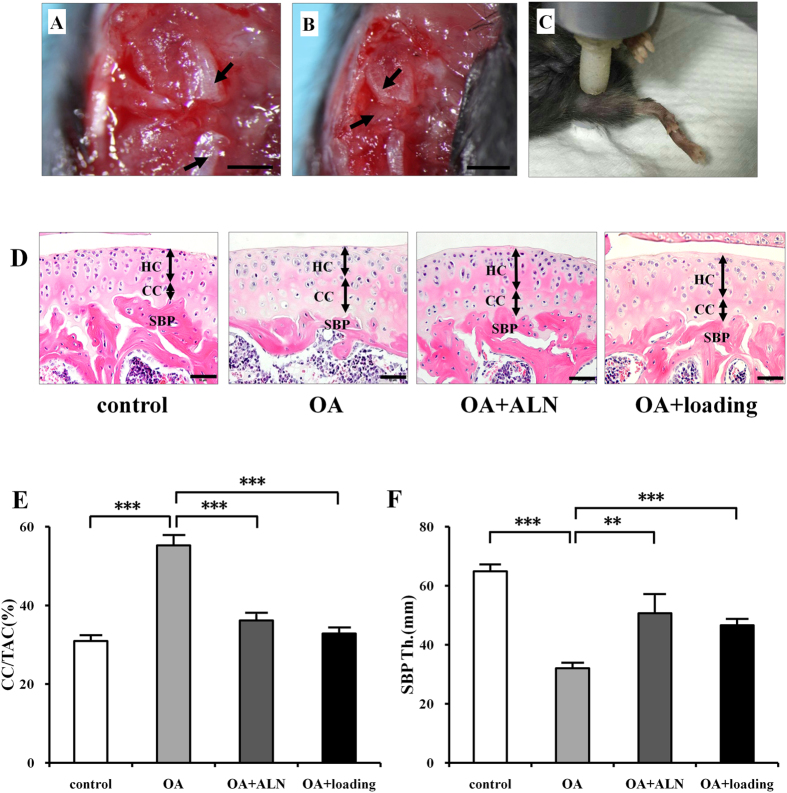
The OA surgery and loading procedure, histological changes of articular cartilage and subchondral bone by H&E staining. (**A**,**B**) The medial collateral ligament was transected (Bar, 1 mm) and the medial meniscus was removed (Bar, 2 mm) on the right knee to induce osteoarthritis. Arrows indicated medial collateral ligament in A, and articular surfaces of femur and tibia in B. (**C**) Daily dynamic knee loading (1 N, 5 Hz) was applied at the right knee 5 min/day for 2 weeks. (**D**) H&E staining of tibial cartilage and subchondral bone plate (SBP). The thickness of hyaline cartilage (HC) and calcified cartilage (CC) were indicated by double arrows. Scale bar, 50 μm. (**E**) Quantitative analysis of the ratio of calcified cartilage to the total articular cartilage (CC/TAC). (**F**) Quantitative analysis of the thickness of subchondral bone plate (SBP). n = 10; **P < 0.01, ***P < 0.001.

To examine the effect of knee loading on articular cartilage and subchondral bone in osteoarthritic mice, the medial collateral ligament (Fig. 1A, arrows) was transected and the medial meniscus (Fig. 1B, arrows showed the articular surfaces of femur and tibia) was removed on the right knee to induce osteoarthritis. Daily dynamic knee loading was applied at 1 N, 5 Hz, 5 min/day for 2 weeks (Fig. 1C). Alendronate (ALN), an anti-resorptive agent, was used as a positive control to evaluate the role of osteoclast activity in OA. The sections were stained with hematoxylin-eosin (H&E). The thickness of the calcified cartilage (CC) increased whereas the hyaline cartilage (HC) layer decreased with tidemark moving closer to articular surface in OA group. The calcified cartilage thickness was attenuated by ALN and loading treatment (Fig. 1D, black double arrows). The ratio of calcified cartilage to the total articular cartilage (CC/TAC) was examined to evaluate the changes of calcified cartilage and hyaline cartilage. Notably, the value of CC/TAC in the OA group was higher than in sham control, OA + ALN, and OA + loading groups (all *P* < 0.001; Fig. 1E). There is no significant difference between knee loading and ALN effect.

**Figure 2:**
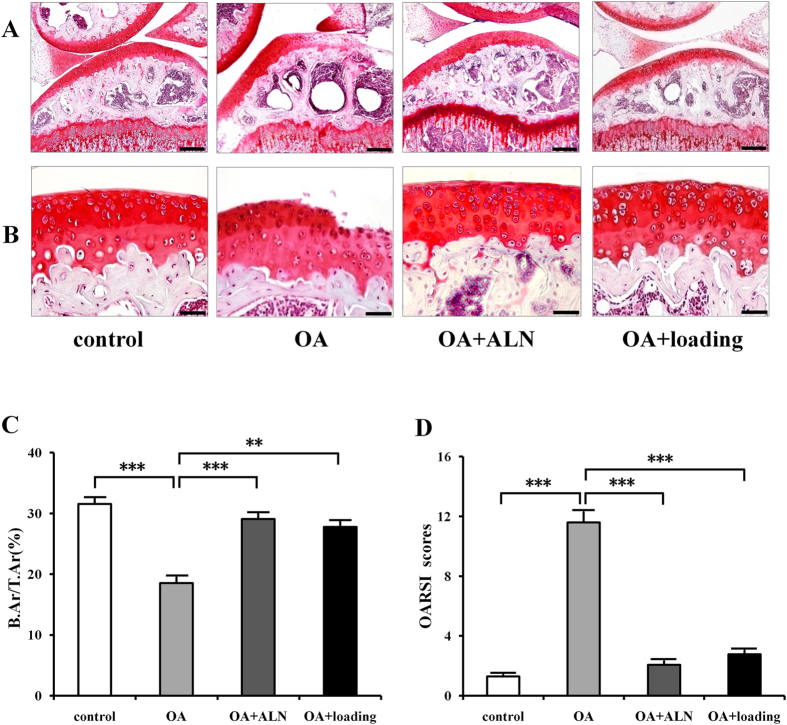
Histological changes of articular cartilage and subchondral bone by Safranin O staining. (**A**) Safranin O staining of tibial subchondral bone. Scale bar, 200 μm. (**B**) Safranin O staining of tibial articular cartilage, red indicates proteoglycan. Scale bar, 50 μm. (**C**) Subchondral bone volume fraction (B.Ar/T.Ar). (**D**) Osteoarthritis development was evaluated by OARSI scores. n = 10; **P < 0.01, ***P < 0.001.

The Safranin O staining showed the surface of the cartilage was smooth in the sham control group; however, it had superficial fibrillation, surface discontinuity, and vertical fissure with apparent hypocellularity in OA group. The OA group also showed massive proteoglycan loss. The cartilage in OA group presented denudation and deformation (Fig. 2B). OARSI scores revealed the significant degeneration of articular cartilage in OA group compared to the sham control (*P* < 0.001). Both knee loading and ALN treatment significantly decreased the OARSI scores (*P* < 0.001; Fig. 2D).

### Histological analysis of subchondral bone

To evaluate the changes of subchondral bone, the thickness of subchondral bone plate (SBP) with H&E staining was measured. The OA group showed a significant thinner in SBP compared to the sham control, OA + ALN, and OA + loading groups (*P* < 0.001). However, the thickness of SBP is significantly increased by treating with alendronate and knee loading (*P* < 0.01, and *P* < 0.001; Fig. 1D,F).

In the sections stained with Safranin O (Fig. 2A), the ratio of trabecular bone area to total tissue area (B.Ar/T.Ar) was detected to evaluate subchondral bone volume fraction. The OA group exhibited a lower B.Ar/T.Ar ratio (*P* < 0.001) compared to the sham control. However, both alendronate and knee loading had significant effects on increasing B.Ar/T.Ar (*P* < 0.001, and *P* < 0.01; Fig. 2C).

In according with histological change in cartilage and subchondral bone of OA by knee loading, the phosphorylation levels of p38 MAPK (p-p38) and NFκB (p-NFκB) were also increased in the development of OA. However, the expression of p-p38 and p-NFκB were significantly decreased by knee loading (Supplementary Fig. S1).

### Changes of osteoclast activity in subchondral bone

**Figure 3:**
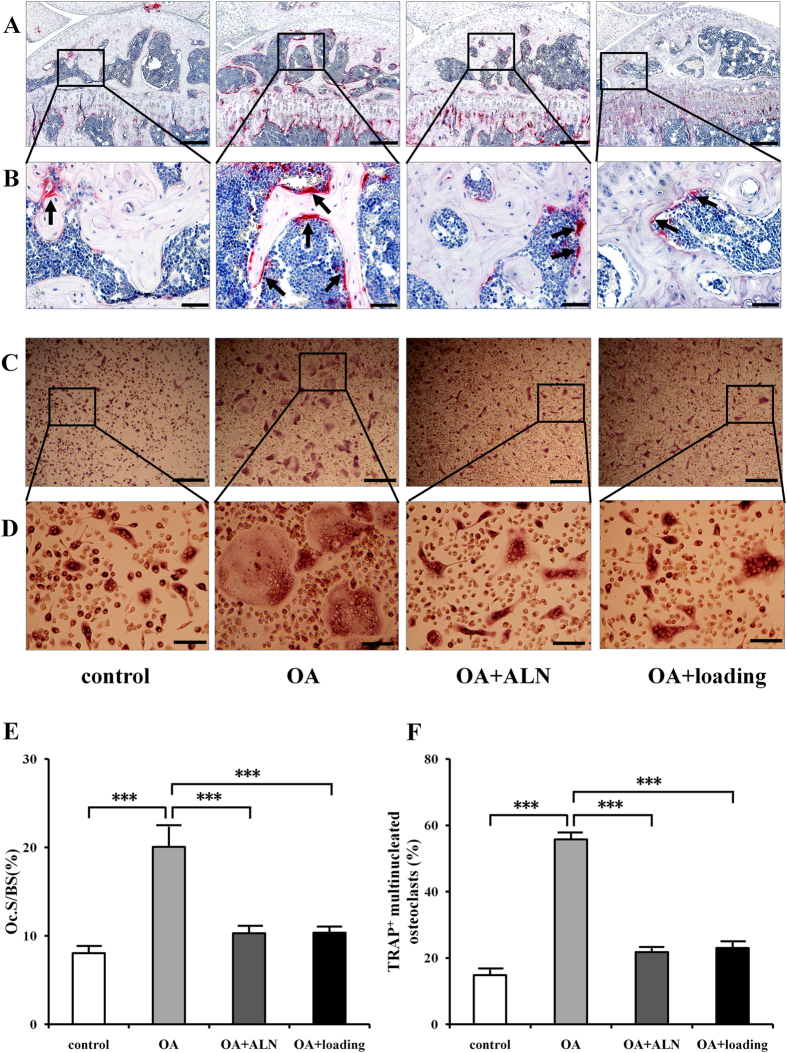
Effects of knee loading on osteoclast activity in subchondral bone and bone marrow-derived cells. (**A**,**B**) TRAP staining of osteoclast in subchondral bone. Red color showed the TRAP-positive cells as osteoclasts in different magnification (**A**) 100 × (Bar = 200 μm), and (**B**) 400 × (Bar = 50 μm). Arrows indicated TRAP-positive cells. (**C,D**) Bone marrow-derived cells were seeded onto 96-well plates at a density of 1 × 10^5^ cells/well. Cells were treated with M-CSF and RANKL for six days. TRAP-positive multinuclear cells (more than 3 nuclei) were identified as mature osteoclasts in different magnification (**C**) 100 × (Bar = 200 μm), and (**D**) 400 × (Bar = 50 μm). (**E**) Quantitative analysis of Oc.S/BS in the trabecular bone. (**F**) Quantitative analysis of the areas covered by TRAP-positive multinucleated osteoclasts. n = 10; ***P < 0.001.

TRAP staining showed that osteoclasts in the subchondral bone were stained red in the surface of trabecular bone (black arrows), and OA mice showed the most TRAP positive cells among these 4 groups (Fig. 3A,B).

Quantified data showed that Oc.S/BS (Osteoclast surface/Bone surface) in the subchondral bone region was significantly increased in the OA group compared to the sham control (*P* < 0.001), which was decreased in the OA + ALN group and OA + loading group (both *P* < 0.001; Fig. 3E).

### Changes of osteoclast formation, migration and adhesion

To determine osteoclast activity *in vitro*, bone marrow-derived cells were harvested and treated with M-CSF and RANKL to induce osteoclast differentiation, and osteoclast lineage (formation, migration, and adhesion) were examined. TRAP staining in osteoclast formation indicated that the maturity and number of osteoclasts in the OA group was the highest among all the four groups (Fig. 3C,D). Quantitative analysis of the area covered by mature osteoclasts showed that bone marrow-derived cells from the OA group exhibited a significantly increased capacity to form matured osteoclasts compared to the sham control (*P* < 0.001). However, knee loading obviously suppressed the formation of matured osteoclasts (*P* < 0.001), and showed no significantly difference compared to the positive control (Fig. 3F).

**Figure 4:**
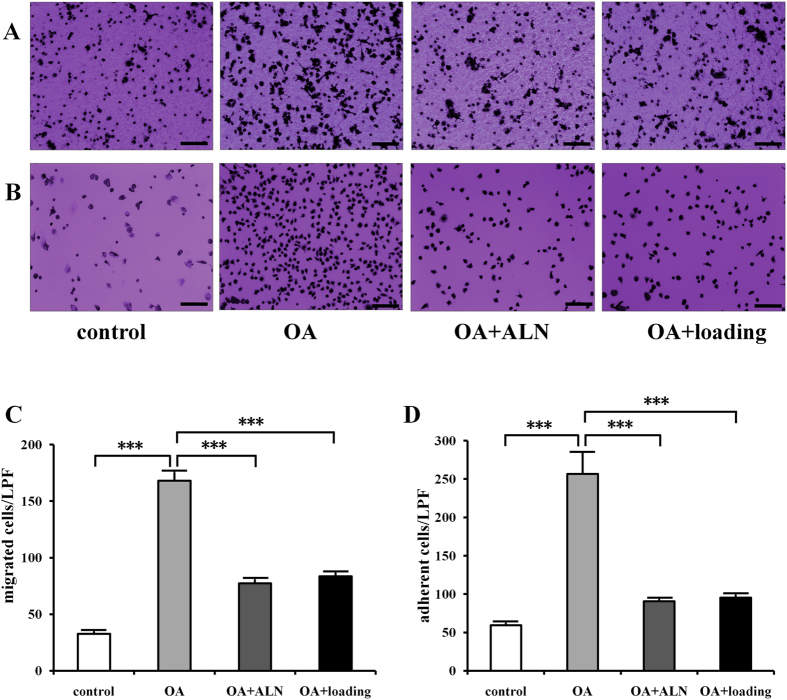
Effects of knee loading on osteoclast migration and adhesion. Bone marrow-derived cells (2 × 10^6^/ml) were cultured with M-CSF and RANKL in 6-well plates for 4 days to obtain pre-osteoclasts used for the migration and adhesion assays. (**A**) Bone marrow-derived cells were treated with M-CSF and RANKL. Then, osteoclast precursors were loaded onto vitronectin-coated polycarbonate membrane of the upper chamber of transwells in 24-well plates at a density of 1 × 10^5^ cells/well at day 4. After 6 h, cells were stained with crystal violet (Bar = 100 μm). (**B**) Bone marrow-derived cells were treated with M-CSF and RANKL for four days. Then osteoclast precursors were plated onto vitronectin-coated 96-well plates at a density of 1 × 10^5^ cells/well. After incubation for 1 h, adherent cells were stained with crystal violet. The number of cells adherent to the bottom of plates was counted (Bar = 100 μm). (**C**) Quantitative analysis of number of migration cells. (**D**) Quantitative analysis of number of adhesion cells. n = 10; ***P < 0.001.

The osteoclast migration (Fig. 4A) and adhesion (Fig. 4B) were conducted to evaluate the effect of knee loading in osteoclast function. The OA group elevated migration and adhesion, while alendronate and knee loading significantly attenuated migration (Fig. 4C) and adhesion (Fig. 4D) (all *P* < 0.001).

### The correlations between osteoclast activity and parameters of cartilage and subchondral bone in OA

**Figure 5:**
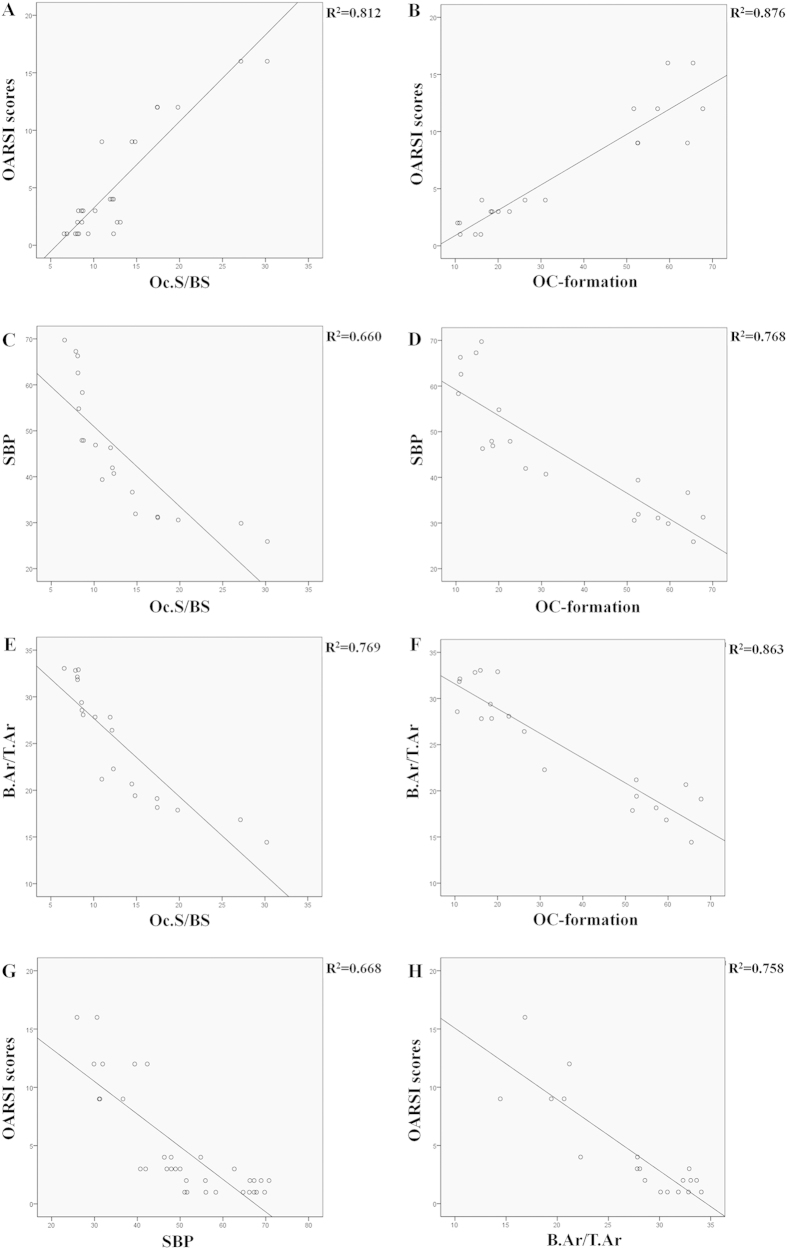
Correlations between osteoclast activity and parameters of cartilage and subchondral bone in OA. Correlations between (**A**) Oc.S/BS, (**B**) osteoclast formation and OARSI scores. Correlations between (**C**) Oc.S/BS, (**D**) osteoclast formation and SBP. Correlations between (**E**) Oc.S/BS, (**F**) osteoclast formation and B.Ar/T.Ar. Correlations between (**G**) SBP, (**H**) B.Ar/T.Ar and OARSI scores. n = 10; ***P < 0.001.

To evaluate the effect of osteoclast on cartilage in osteoarthritis the correlations between osteoclast activity and OARSI scores were analyzed. The activity of osteoclasts in the subchondral bone region, osteoclast formation from bone marrow-derived cells were positively correlated with OARSI scores which indicated histopathology of cartilage in osteoarthritis (r = 0.901, 0.936, *P* < 0.001) (Fig. 5A,B).

The correlations between osteoclast activity and parameters of subchondral bone were analyzed to evaluate the effect of osteoclast on subchondral bone in osteoarthritis. The osteoclasts in the subchondral bone region and osteoclast formation were negatively correlated with subchondral bone plate (SBP) (r = −0.812,−0.876, *P* < 0.001) (Fig. 5C,D), and also negatively correlated with the ratio of trabecular bone volume to total tissue volume (B.Ar/T.Ar) (r = −0.877,−0.929, *P* < 0.001) (Fig. 5E,F).

The correlations between SBP, B.Ar/T.Ar and OARSI scores were analyzed to evaluate the link between subchondral bone and cartilage. Both SBP and B.Ar/T.Ar were negatively correlated with OARSI scores (r = −0.818,−0.871, *P* < 0.001) (Fig. 5G,H).

### The multiple linear regression models for osteoclast activity and parameters of cartilage and bone

The multiple linear regression models between the osteoclasts formation, Oc.S/BS and OARSI scores, SBP and B.Ar/T.Ar were generated to analysis potential predictors of changes in OA (Table 1). The multiple linear regression models indicated osteoclast activity is the predictor of the changes of OARSI scores, SBP, B.Ar/T.Ar and these models could guide the prediction effectively.

The correlations between osteoclast activity and OARSI scores, SBP, B.Ar/T.Ar showed that they were matched to each other (R^2^ = 0.918, 0.896, 0.889 respectively, *P* < 0.001, n = 30). The osteoclasts formation, Oc.S/BS were positively associated with OARSI scores, and negatively associated with SBP and B.Ar/T.Ar.

### The expression of Wnt3a and osteoclastogenesis genes including NFATc1, RANKL, TNF-α, and Cathepsin K

**Figure 6:**
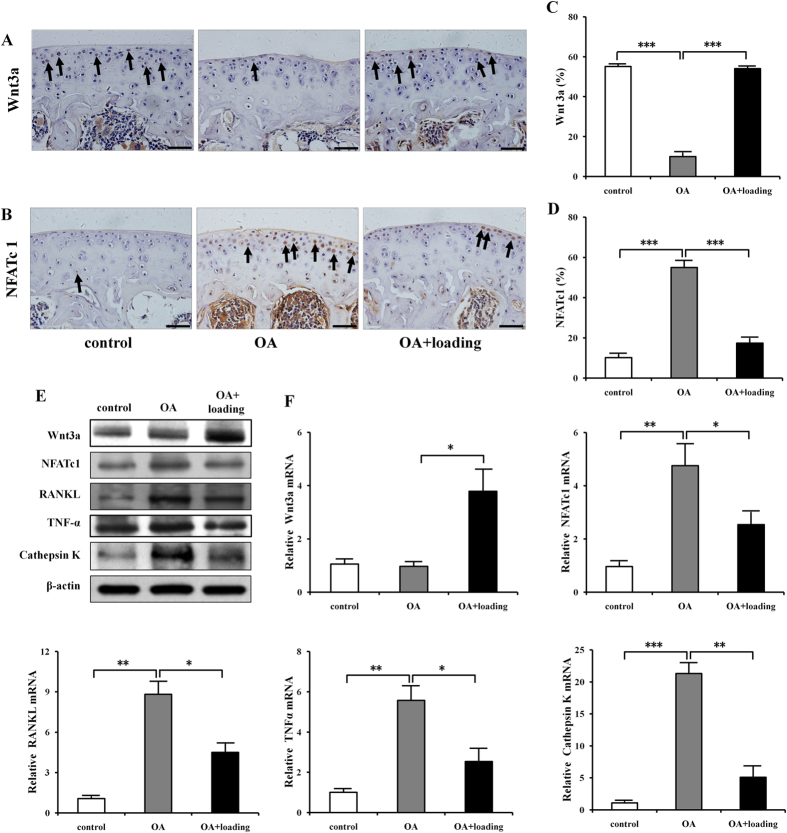
Effects of knee loading on gene expression in a mouse model of osteoarthritis. (**A,B**) Immunohistochemistry staining of Wnt3a and NFATc1 in sagittal sections of tibial cartilage were conducted (Bar = 50 μm). (**C,D**) Quantification of Wnt3a and NFATc1-positive cells in tibial cartilage were examined. n = 10. (**E**) Western blot analysis of Wnt3a, NFATc1, RANKL, TNF-α, and Cathepsin K in OA treated with knee loading. Cropped blots were used in the figure. Full-length blots were presented in Supplementary Fig. S2. (**F**) Relative mRNA levels of Wnt3a, NFATc1, RANKL, TNF-α, and Cathepsin K. n = 6; *P < 0.05, **P < 0.01, ***P < 0.001.

To evaluate the mechanism of knee loading effect on OA at the molecular level, we examined the activities of Wnt3a, together with NFATc1, RANKL, TNF-α, and Cathepsin K in the tibia. Immunohistochemistry staining of Wnt3a and NFATc1 further validated the effects of knee loading on promotion of Wnt3a and inhibition of NFATc1 in cartilage cells (Fig. 6A–D). Both western blot analysis and quantitative real-time PCR of proximal tibia showed that the expression of Wnt3a was significantly increased by knee loading. However, the protein and mRNA levels of NFATc1, RANKL, TNF-α, and Cathepsin K were significantly suppressed by knee loading (Fig. 6E,F and Supplementary Fig. S2).

## Discussion

This study demonstrated that knee loading reduced OARSI score, attenuated the ratio of calcified cartilage to the total articular cartilage (CC/TAC), and increased thickness of the subchondral bone plate and subchondral B.Ar/T.Ar in a mouse model of surgery-induced osteoarthritis. These findings verified the effects of knee loading on articular cartilage and subchondral bone of osteoarthritis. The data also showed that the activity of osteoclasts was significantly increased in OA mice not only in the subchondral bone region but also in differentiation from bone marrow-derived cells. Furthermore, correlation analysis showed a significant positive correlation between osteoclast activity and OARSI scores. The activity of osteoclasts in the subchondral bone and bone marrow-derived cells was significant negatively correlated with subchondral bone plate (SBP) and the ratio of trabecular bone volume to total tissue volume (B.Ar/T.Ar). Otherwise, the multiple linear regression models indicated increasing osteoclast formation and Oc.S/BS associated with the increase in OARSI scores and decrease of SBP and B.Ar/T.Ar.

Cartilage degeneration and structure of subchondral bone changes are main features of OA^27^. OARSI scores was an OARSI Working Group deliberated on principles, standards and features for an OA cartilage pathology assessment system^28^,^29^. Our study showed that these changes of OARSI score reduced by knee loading coincide with the prevention of cartilage degeneration. Increase of SBP and B.Ar/T.Ar by knee loading indicated that mechanical loading enhanced thickness and trabecular bone volume fraction in subchondral bone. These data suggested that mechanical knee loading regulates both cartilage and subchondral bone of OA. Joint loading is a non-invasive physical treatment applied to synovial joints for orthopedic diseases^19^. Our previous data found that knee loading stimulates bone formation, and accelerates the healing of surgical wounds in the tibia and femoral neck^20^,^23^. In OA, MMP13 expression in the femoral cartilage is also reduced by knee loading^24^. Other studies showed that moderate mechanical stimuli including aerobic exercise prevent cartilage degeneration, and treadmill running exercise affect on subchondral and trabecular bone metabolism such as subchondral bone cysts growth and osteocyte death, and protect cartilage degeneration in surgery-induced OA or chemical-induced OA model^14^,^30^,^31^,^32^. Mechanical loading is an important factor in prevent subchondral bone remodeling and cartilage damage, but the underlying mechanism remains unclear.

Many studies focus on the interaction among cartilage, bone, bone marrow in OA. Subchondral bone and mesenchymal stem cells play crucial roles in the initiation and progression of cartilage degeneration in osteoarthritis through bone remodeling involved the osteoclast activity^33^,^34^,^35^. In the current study, a positive correlation between cartilage degeneration indicated by OARSI scores and osteoclasts from both subchondral bone and bone marrow derived cells, suggested that links between cartilage and osteoclast activity. The negative correlation between SBP, B.Ar/T.Ar and OARSI score indicated relationship of subchondral bone and cartilage. The crosstalk bone-cartilage exists not only by form a complex functional unit^10^ but also by osteoclast activity. Subchondral bone thickness and bone volume fraction were significantly negative correlated with osteoclasts from TRAP-stained histology sections and bone marrow-derived cells, indicating subchondral bone remodeling is associated with osteoclast activity. The changes in subchondral bone are closely related to osteoclast activity. Subchondral bone cysts growth as an obvious characteristic of bone adaptation associated with osteoarthritis is produced by osteoclast activity in early osteoarthritis^11^,^36^.

**Figure 7:**
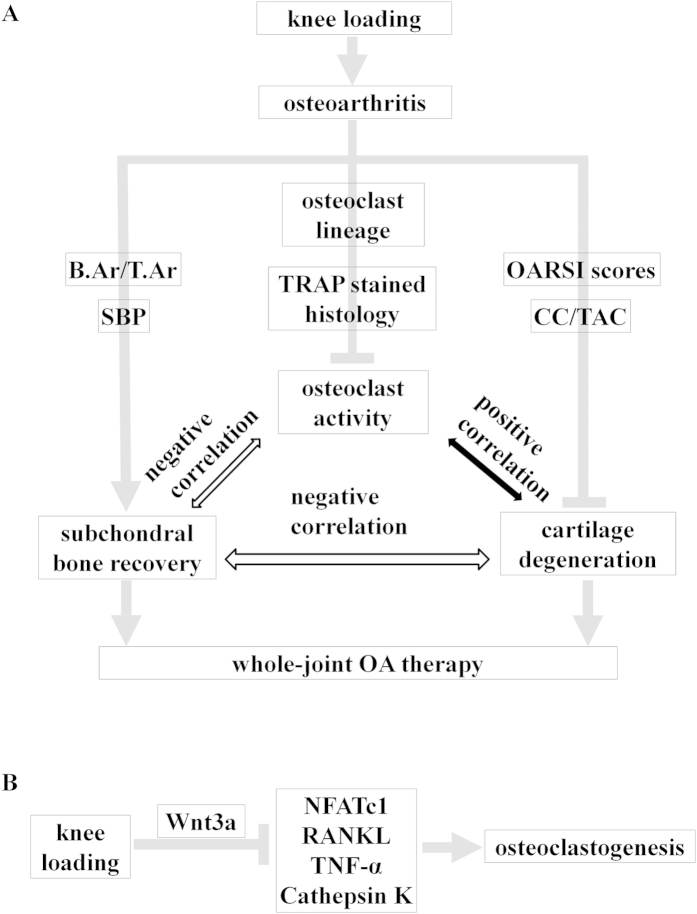
Proposed mechanism of knee loading affects on OA by inhibiting osteoclast development. (**A**) A significant positive correlation was observed between osteoclast activity and cartilage degeneration. A significant negative correlation was observed between osteoclast activity and subchondral bone recovery. Subchondral bone recovery negatively correlated with cartilage degeneration. (**B**) Proposed molecular mechanism of knee loading suppresses osteoclastogenesis by Wnt signaling pathway.

Furthermore, we also generated the multiple linear regression models to confirm osteoclast activity as a crucial factor for osteoarthritis. The mathematic models showed that increasing osteoclast formation and Oc.S/BS for osteoclast activity assessment, consistent with the increase in OARSI scores as evaluation of cartilage degeneration and decrease of SBP and B.Ar/T.Ar as structure changes of subchondral and trabecular bone. The multiple linear regression models showed osteoclast activity are significant predictors for the changes in OA, including prevented cartilage degradation and regulated subchondral bone remodeling. Osteoclasts originate from haemopoietic stem cells and activated osteoclasts are multinucleated giant cells for bone resorption involved as one of the two factors for bone remodeling^37^,^38^. Inhibiting osteoclatic bone resorption has been reported in animal models of osteoarthritis treated with bisphosphonates such as alendronate and zolendronic acid^39^,^40^,^41^. To verify the role of osteoclast activity in cartilage and subchondral bone, alendronate, which is an anti-osteoclastic bone resorption agent, was used as a positive control in OA. The results showed that knee loading as well as alendronate could decrease the OARSI scores and increase B.Ar/T.Ar, meanwhile, inhibit Oc.S/BS and osteoclast formation, migration, and adhesion. Therefore, the preventive effects of knee loading on cartilage and subchondral bone might be mainly explained by suppressing the osteoclast activity (Fig. 7A).

Wnt/β-catenin signaling is one of the pathways that are normal response to mechanical loading in bone cells^25^,^26^,^42^. Here, we investigated whether knee loading suppressed osteoclastogenesis by Wnt signaling pathway. The results indicated that mechanical loading increased the expression of Wnt3a, and reduced expression of NFATc1 (a master transcription factor for development of osteoclasts), RANKL, TNF-α, and Cathepsin K. We had previously shown that Wnt signaling inhibited osteoclastogenesis by decreased the expression of genes that were involved in osteoclast development^43^. Thus, knee loading might suppress osteoclastogenesis by Wnt signaling pathway in the treatment of OA (Fig. 7B). Wnt/β-catenin signaling as a crucial role for osteoclast bone resorption was also certified by β-catenin inactivation and deletion increased osteoclast number and activity to promote bone loss^44^,^45^,^46^.

In conclusion, these data have shown that knee loading is effective in decrease OARSI score and CC/TAC, increase SBP and B.Ar/T.Ar as with ALN in OA animal model. The results demonstrate that knee loading has a tendency to prevent cartilage degradation and regulate subchondral bone remodeling by suppressing abnormal osteoclast activity in the knee osteoarthritis. Osteoclast activity plays a significant role in the interaction between articular cartilage and subchondral bone, and knee loading might suppresses osteoclastogenesis through Wnt signaling pathway in the treatment of osteoarthritic mice. Therefore, knee loading which inhibits osteoclast activity may be a new possible non-invasive strategy for OA treatment. Future studies should focus on the underlying molecular mechanism of the effect of osteoclast activity and knee loading in OA.

## Methods

### Animals and materials preparation

All experiments were carried out according to the National Institutes of Health Guide for Care and Use of Laboratory Animals and approved by the Ethics Committee of the Tianjin Medical University. Female C57BL/6 mice (~14 weeks of age) were purchased from Animal Center of Academy of Military Medical Sciences (China). Five mice per cage were fed with mouse chow and water *ad libitum*. The mice acclimatized to the 12:12 h light-dark cycle conditions in the cages. Cytokines were purchased from PeproTech (Rocky Hills, NC, USA). Medium, fetal bovine serum, penicillin, streptomycin and trypsin were purchased from Invitrogen (Carlsbad, CA, USA). Other chemicals were purchased from Sigma (St. Louis, MO, USA).

### Osteoarthritis surgery

Sixty-four mice were divided into 4 groups randomly (n = 16): sham control (control), osteoarthritis (OA), alendronate treated OA (OA + ALN), and loaded osteoarthritis (OA + loading). The mouse was anesthetized with 1.5% isoflurane (IsoFlo, Abbott Laboratories, North Chicago, IL, USA) at a flow rate of 1.0 L/min, and the right hindlimb was shaved and sterilized with 70% alcohol solution. Using a scalpel, a 20 mm skin incision was made to expose the right knee joint and the medial collateral ligament was transected. After articular cavity was opened with scalpel, the medial meniscus was removed using a surgical microscope and microsurgical technique^24^. Then irrigation with saline to remove tissue debris, the skin incision was closed. For the sham control, the surgery was performed on the right knee using the same approach without the ligament transection and the medial meniscus remove. Buprinorphine hydrochloride was conducted as analgesia after surgery.

### Knee loading

The OA + loading mice were subject to knee loading. At one week after surgery, knee loading was applied to the right hindlimb using a custom-made loader for 2 weeks^19^,^20^. During loading process, the mouse was mask-anesthetized using 1.5% isoflurane. Dynamic loads were sinusoidal at 1 N (peak-to-peak) and the frequency was 5 Hz for 5 min/day. The loading device was calibrated using a load cell (Model LLB130, FUTEK Advanced Sensor Technology, Irvine, CA) to determine peak compressive force for increasing actuator voltage. To position the knee properly, the upper end of the stator and the lower end of the loading rod were designed to form a semispherical cup. The lateral and medial epicondyles of the femur together with the lateral and medial condyles of the tibia were confined in the cup^47^. The tip of the loader diameter was a 5 mm contact area^48^. The sham control and OA mice were placed on the loading device without any dynamic force after anesthetized. The right knees were harvested for histological analysis, and bone marrow-derived cells were isolated.

### Subcutaneous administration of alendronate

To evaluate the role of osteoclast activity in OA treatment, alendronate which is one of bisphosphonates was used as a positive control. Sixteen OA mice received subcutaneous injections of alendronate (Sigma, St. Louis, MO, USA) in PBS daily at a dose of 300 μg/kg for 2 weeks. The placebo mice received an equal volume of vehicle solution (PBS)^39^,^41^.

### Histology and immunohistochemistry assay

Knee samples were fixed in 10% neutral buffered formalin for 2 days, and decalcified in 14% Ethylene Diamine Tetraacetic Acid (EDTA) for 2 weeks. Decalcified samples were embedded in paraffin, and the samples were sectioned at 5 μm thickness along the sagittal plane. Slides were stained with H&E for observing the histological parameters of articular cartilage. Safranin O staining of tibial cartilage was performed to evaluate the effect of knee loading on osteoarthritic mice. Measurements were performed area on the proximal side of the growth plate, in which B.Ar/T.Ar (in %, T.Ar, total tissue area, calculated from the total tissue area; and B.Ar, trabecular bone area, calculated from the total trabecular area) was determined^46^. The number of subchondral bone osteoclasts was evaluated by tartrate resistant acid phosphatase (TRAP) staining. Oc.S/BS was calculated. It represented the activity of osteoclasts. The images obtained by an Olympus CCD DP73 (Olympus) were processed using CellSense Standard software (Olympus). For immunohistochemical analysis, sagittal sections of knee joint were incubated with primary antibodies against Wnt3a (Abcam, Cambridge, MA, USA) and NFATc1 (Proteintech, Wuhan, China) at 4 °C overnight. Immunohistochemical kit and 3, 3′-diaminobenzidine (DAB) (ZSGB-BIO Corporation, China) substrate kit were used according to the protocol of manufacturer. Histomorphometric measurements were conducted on the area of the tibial cartilage. The number of positive stained cells was counted in the whole tibial cartilage area per specimen and five sequential specimens per mouse in each group were measured. Quantitative analysis was conducted in a blinded fashion^47^.

### Histopathological grade assessment of osteoarthritis cartilage

Tissue sections were stained with Safranin O staining, and graded by Osteoarthritis Research Society International (OARSI) score. Score is defined as assessment of combined grade and stage of OA, and represents a combined assessment of severity and extent of OA. Section was assigned as follows six OA grades: 0 = surface intact, cartilage intact; 1 = surface intact; 2 = surface discontinuity; 3 = vertical fissures; 4 = erosion; 5 = denudation; 6 = deformation. Four OA stages, 1 = 10% horizontal extent of the involved cartilage surface irrespective of underlying OA grade; 2 = 10–25% involvement; 3 = 25–50% involvement; 4 = more than 50% involvement^28^.

### Isolation of bone marrow-derived cells and osteoclast formation

Using a 23-gauge needle, bone marrow-derived cells were collected by flushing the iliac with Iscove’s MEM (Gibco-Invitrogen, Carlsbad, CA, USA) containing 2% fetal bovine serum (FBS)^47^,^49^. Cells were separated by low-density gradient centrifugation and cultured in α-MEM supplemented with 10% FBS, 30 ng/ml murine macrophage-colony stimulating factor (M-CSF), and 20 ng/ml murine receptor activator of nuclear factor kappa-B ligand (RANKL). On day 4, the culture medium was replaced by α-MEM supplemented with 10% FBS, 30 ng/ml M-CSF, and 200 ng/ml RANKL, and cells were grown for an additional 3 days^49^,^50^. Cells were fixed and stained with a tartrate resistant acid phosphate (TRAP)-staining kit according to the manufacturer’s instructions. TRAP-positive multinucleated cells (>3 nuclei) were identified as mature osteoclasts, and the area covered by mature osteoclasts was determined^51^.

### Osteoclasts migration and adhesion

Migration of osteoclasts was evaluated using a transwell assay as described previously with minor modifications^48^. Bone marrow-derived cells (2 × 10^6^ cells/ml in 6-well plates) were cultured in M-CSF and RANKL for 4 days. Then α-MEM was used to wash non-attaching cells. TRAP staining was conducted to identify that the remaining mononucleated cells were osteoclast precursors. The osteoclast precursor cells (1 × 10^5^ cells/well) were loaded onto the upper chamber of transwells and allowed to migrate to the bottom chamber through an 8-μm polycarbonate filter coated with vitronectin (Takara Bio Inc., Otsu, Sigma, Japan). The bottom chamber contained α-MEM consisting of 1% bovine serum albumin (BSA) and 30 ng/ml M-CSF. After 6 h reaction, the number of osteoclast precursor cells in the lower chamber (attached onto the bottom of the transwells) was stained with crystal violet and counted.

For assaying adhesion, osteoclast precursor cells (1 × 10^5^ cells/well) were placed into 96-well plates coated with 5 μg/ml vitronectin in α-MEM supplemented with 30 ng/ml M-CSF^52^. After 30 min of incubation, cells were washed with PBS three times and fixed with 4% paraformaldehyde at room temperature for 10–15 min. Cells were stained with crystal violet, and the number of cells adherent to the bottom of plates was counted.

### Western blot analysis

Three weeks after the induction of OA, mice (n = 6 for each group) were sacrificed for western blot analysis. Protein samples were isolated from proximal tibia using a mortar and pestle. Tissues were lysed in a radioimmunoprecipitation assay (RIPA) lysis buffer, containing protease inhibitors and phosphatase inhibitors (Roche Diagnostics GmbH, Mannheim, Germany). Isolated proteins were fractionated using 10% sodium dodecyl sulfate-polyacrylamide gel and electro-transferred to polyvinylidene difluoride membranes (Millipore, Billerica, MA, USA). Primary antibodies specific to Wnt3a (Abcam, Cambridge, MA, USA), together with NFATc1, RANKL, TNF-α, Cathepsin K, p38 MAPK, NFκB p65 (Rel A) (Proteintech, Wuhan, China), Phosph-p38 MAPK, Phosph-NFκB (Cell Signaling, Danvers, MA, USA) and β-actin (Sigma, St Louis, MO, USA) were employed. After incubation with secondary IgG antibodies conjugated with horseradish peroxidase, signals were detected with enhanced chemiluminescence. Data were presented with reference to control intensities of β-actin^24^.

### Quantitative real-time PCR

Total RNA was extracted from proximal tibia using RNA Prep Pure Tissue Kit (TIANGEN Biotech, Beijing, China). First-stranded cDNA was synthesized by RevertAid First Strand cDNA Synthesis Kit (Thermo Scientific, Waltham, MA, USA). Quantitative real-time PCR was performed on IQ5 (Bio-Rad, Hercules, CA, USA) using Power SYBR Green PCR Master Mix (Thermo Scientific, Waltham, MA, USA). Target genes Wnt3a (forwad: 5′-TAC CCG ATC TGG TGG TCC TT-3′, and reverse 5′-GGG CAT GAT CTC CAC GTA GT-3′), NFATc1 (forwad: 5′-CTA TCG AGT GTT CCC AGC GG -3′, and reverse 5′-AGT TAT GGC CAG ACA GCA CC-3′), RANKL (forwad: 5′-CAT CCC ATC GGG TTC CCA TA-3′, and reverse 5′-GTC TGT AGG TAC GCT TCC CG-3′), TNF-α (forwad: 5′- ACA GAA AGC ATG ATC CGC GA-3′, and reverse 5′-GGG AAC TTC TCA TCC CTT TGG G-3′), Cathepsin K (forwad: 5′-CTG TGG AGG CGG CTA TAT GAC-3′, and reverse 5′-ACT TTC ATC CTG GCC CAC AT-3′) was normalized to the reference gene β-actin^24^.

### Statistical analysis

The data were expressed as mean ± SEM. Statistical significance among groups was examined using one-way ANOVA. And a post-hoc test of least significant difference tests. Use Pearson correlation coefficient test for correlation analysis. The multiple linear regression models were used to determine potential predictors of changes in OA. All comparisons were two-tailed and statistical significance was assumed for *P* < 0.05. The single, double and triple asterisks in figures indicate *P* < 0.05, *P* < 0.01 and *P* < 0.001, respectively.

## Additional Information

**How to cite this article**: Li, X. *et al.* Knee loading inhibits osteoclast lineage in a mouse model of osteoarthritis. *Sci. Rep.* **6**, 24668; doi: 10.1038/srep24668 (2016).

## Supplementary Material

Supplementary Information

## Figures and Tables

**Table 1:** Multiple linear regression models for significant predictors of osteoclast (OC) -formation, and Oc.S/BS.

	β Coefficient	95% Confidence interval	*p*-value
OARSI scores (R^2^ = 0.918, n = 30)			
OC-formation	0.119	0.067 to 0.170	0.000
Oc.S/BS	0.403	0.218 to 0.587	0.000
SBP (R^2^ = 0.896, n = 30)			
OC-formation	−0.425	−0.570 to −0.280	0.000
Oc.S/BS	−0.859	−1.322 to −0.395	0.001
B.Ar/T.Ar (R^2^ = 0.889, n = 30)			
OC-formation	−0.139	−0.206 to −0.072	0.000
Oc.S/BS	−0.547	−0.761 to −0.333	0.000
