# Supplementary material for: Knee loading inhibits osteoclast lineage in a mouse model of osteoarthritis
Source: Sci Rep. 2016 Apr 18;6:24668. doi: 10.1038/srep24668 (PMC4834538; doi:10.1038/srep24668)
Supplement: Supplementary Information [file srep24668-s1.doc]

**Supplementary Information**

**Knee loading inhibits osteoclast lineage in a mouse model of osteoarthritis**

**Xinle Li^1^, Jing Yang^1^, Daquan Liu^1,2^, Jie Li^1^,** **Kaijun Niu^3^, Shiqing Feng^4^, Hiroki Yokota^5^, and Ping Zhang^1,5,6^***

**Supplementary Figure 1**

**
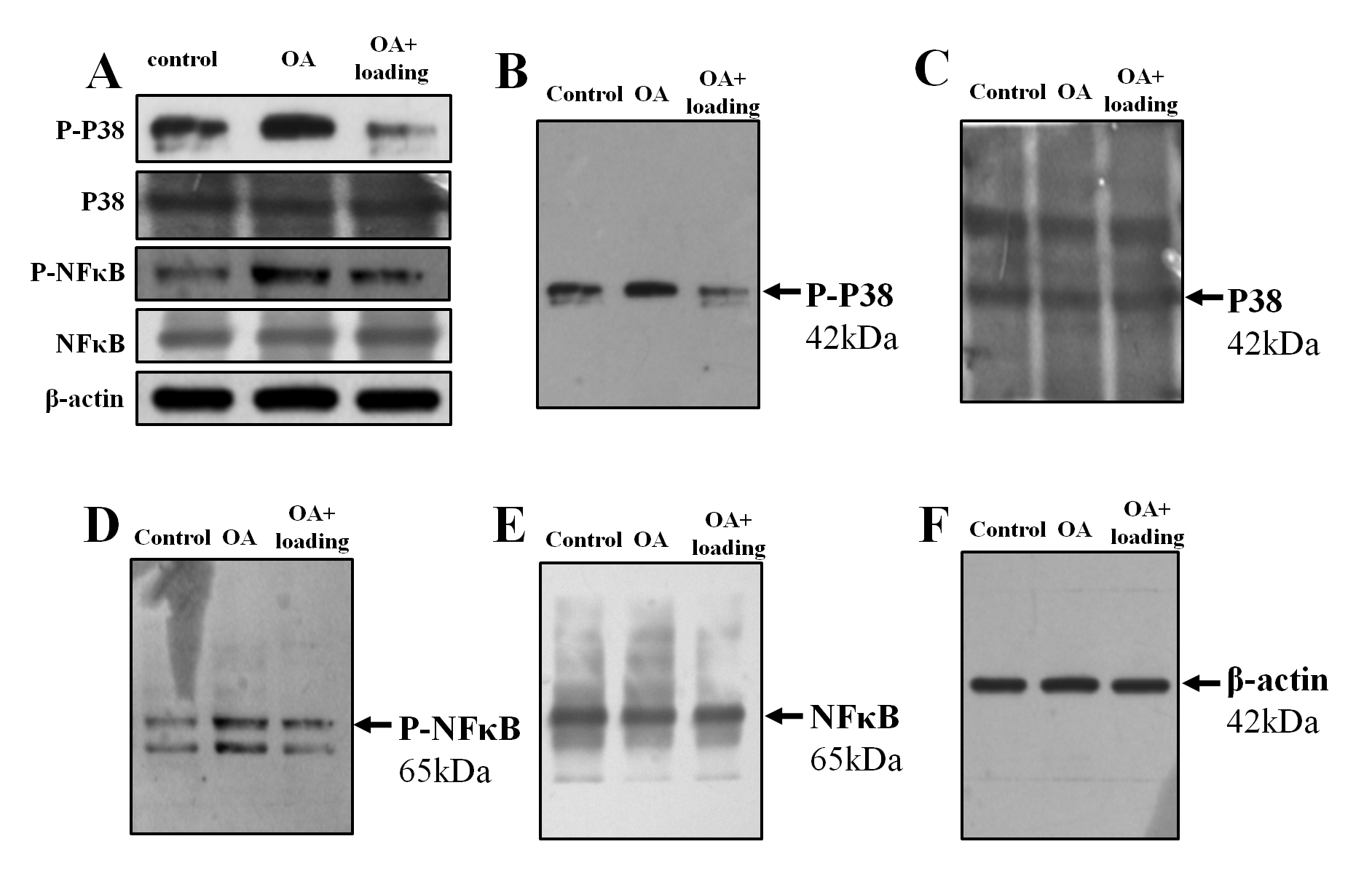
**

**Supplementary Figure S1: Effects of knee loading on the phosphorylation levels of p38 MAPK and NFκB in OA mice.** (A) Phosphorylation levels of p38 MAPK (p-p38) and NFκB (p- NFκB) in OA and knee loading. (B-F) Full-length blots of (B) P-P38, (C) P38, (D) P-NFκB, (E) NFκB, and (F) β-actin by western blot analysis. n = 6.

**Supplementary Figure 2**


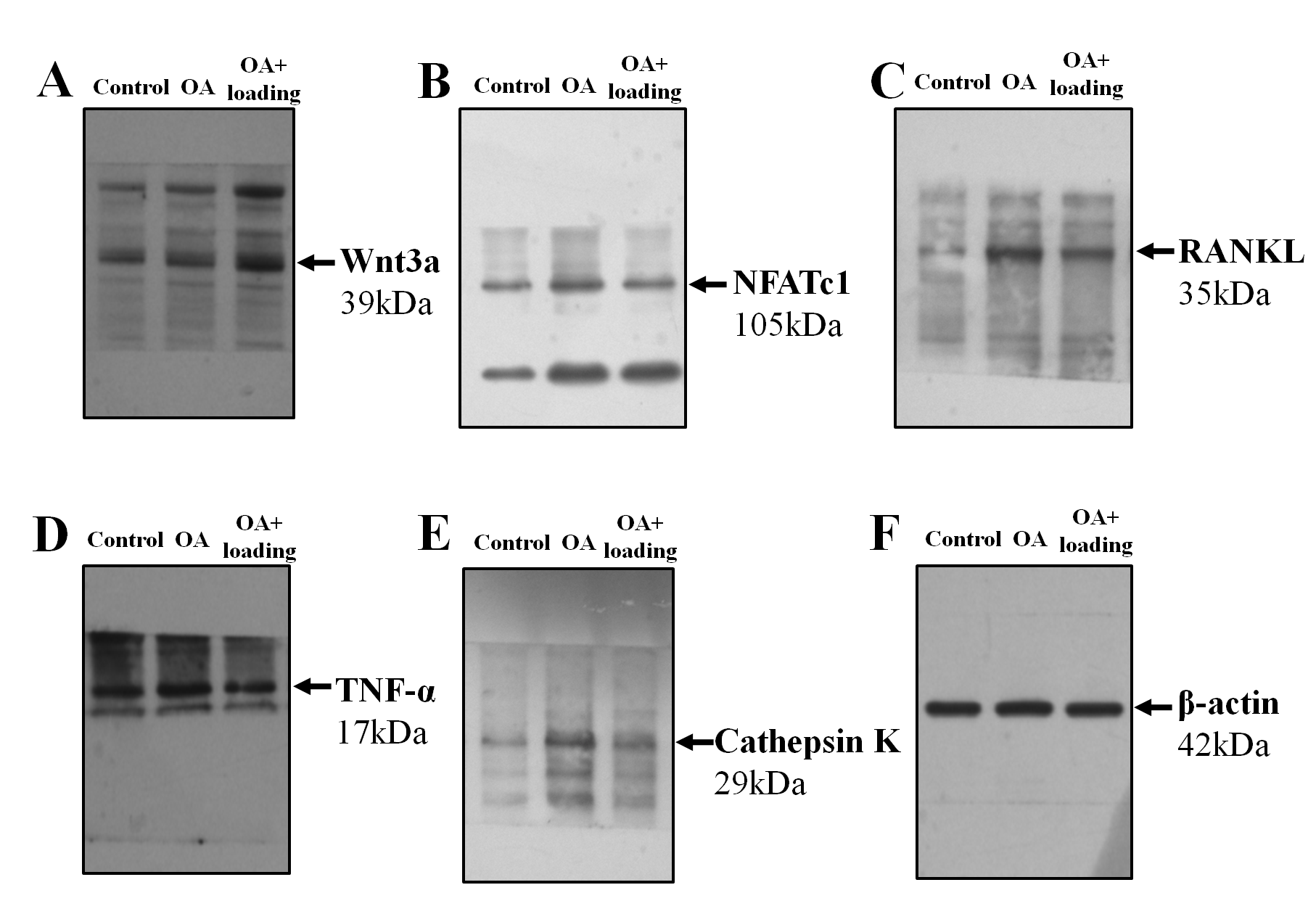


**Supplementary Figure S2: Full-length blots of western blot analysis.** (A) Wnt3a, (B) NFATc1, (C) RANKL, (D) TNF-α, (E) Cathepsin K, and (F) β-actin expression in loading OA showed by full-length blots of western blot. n = 6.
